# Combining Video‐Based Skeletal and Wearable Physiological Data for Early Detection of Agitation in Dementia Using Deep Learning

**DOI:** 10.1002/alz70857_105825

**Published:** 2025-12-25

**Authors:** Somayya Elmoghazy, Sara Elgazzar, Abeer Badawi, Khalid Elgazzar, Amer M. Burhan

**Affiliations:** ^1^ Ontario Tech University, Oshawa, ON, Canada; ^2^ University of Toronto, Toronto, ON, Canada; ^3^ Ontario Shores Centre for Mental Health Sciences, Whitby, ON, Canada

## Abstract

**Background:**

Agitation and aggression (AA) in dementia pose significant challenges for patients, caregivers, and healthcare providers. Current detection methods rely on direct observation, delaying intervention and increasing inappropriate psychotropic medication use. Technological approaches, such as actigraphy and multimodal sensory wearables, have shown promise but remain limited in sensitivity and specificity. Advances in deep learning provide new opportunities to enhance early detection by integrating multimodal data sources.

**Methods:**

This study employs a fusion of video‐based skeletal keypoints and wearable‐driven physiological data to improve AA prediction in individuals with advanced dementia in an inpatient setting. Physiological data were collected using the EmbracePlus wristband, which captures raw signals and digital biomarkers. Concurrently, privacy‐compliant software extracted skeletal keypoints from video footage, ensuring patient anonymity. A standardized preprocessing pipeline aligned both data sources through sampling, normalization, and feature extraction.

A deep learning framework incorporating recurrent neural networks (RNN) with gated recurrent units (GRU) and attention mechanisms was implemented to identify temporal patterns associated with agitation episodes.

**Results:**

Preliminary analysis includes five patients (three females, two males; ages 63–85) with Alzheimer's or mixed pathology dementia and AA. Data collection per participant ranged from 36 to 95.5 hours. Model performance demonstrated an accuracy of 0.98 when integrating both video and wearable data, outperforming single‐modality inputs (video: 0.94, wearable: 0.91).

**Conclusions:**

Integrating wearable physiological data with video‐derived skeletal keypoints enhances the early detection of agitation in dementia care units. This approach is feasible, privacy‐compliant, and improves predictive accuracy. Further research is underway to refine and scale this model for broader clinical application.

